# Validating the General Extended Technology Acceptance Model for E-Learning: Evidence From an Online English as a Foreign Language Course Amid COVID-19

**DOI:** 10.3389/fpsyg.2021.671615

**Published:** 2021-10-01

**Authors:** Michael Yi-chao Jiang, Morris Siu-yung Jong, Wilfred Wing-fat Lau, Yan-li Meng, Ching-sing Chai, Mengyuan Chen

**Affiliations:** ^1^Department of Curriculum and Instruction, Faculty of Education, The Chinese University of Hong Kong, Hong Kong, SAR China; ^2^Centre for Learning Sciences and Technologies, Faculty of Education, The Chinese University of Hong Kong, Hong Kong, SAR China; ^3^Faculty of Foreign Language Teaching, Shenyang Normal University, Shenyang, China

**Keywords:** technology acceptance model, GETAMEL, validation, online EFL course, extended technology acceptance model

## Abstract

The present study validated the general extended technology acceptance model for e-learning (GETAMEL) with the survey data from the English as a foreign language (EFL) online class during the novel coronavirus lockdown period. A total of 678 undergraduates participated in the survey. Structural equation modeling was employed to analyze the data. The results showed that the influence of perceived usefulness of students on their intentional behavior to use the online learning system was not mediated by their attitude, indicating a very limited role of attitude toward technology in the model. Enjoyment and self-efficacy had no significant effects on the internal constructs, raising theoretical concerns on the applicability of this general model into specific contexts. In addition, we found that experience might be a moderator rather than an antecedent of the internal constructs in the model.

## Introduction

Technology acceptance is a critical perspective in an educational context to understand the acceptability of new technology. This is not only because the development of educational technology has never ceased, but also because some social events may generate new demands. On March 11, 2020, the WHO declared COVID-19 a pandemic (World Health Organization., 2020), which created a huge demand for online learning. In response, school education at all levels worldwide began shifting from offline classrooms to fully online instruction. Consequently, teachers were “forced” to become network anchors and livestream their lectures, and students had to complete all the courses online. In China, although online education has been carried out for years (e.g., Xie et al., 2001; Liu et al., 2003), many students have never had any experience of formal online learning in school education. Under the influence of the COVID-19 pandemic, maintaining an acceptable standard of learning in a fully online context for the student population has become a major concern for teachers and educational institutions.

The past three decades have witnessed the emergence of some theoretical models to investigate technology acceptance and use, among which the technology acceptance model (TAM) is one of the most widely applied models (Agudo-Peregrina et al., 2014). Based on the TAM, several studies have attempted to integrate constructs from competing models (Venkatesh et al., 2003) that can influence users' perception of new technologies, and thus extending the TAM models (e.g., Liu, 2010; Teo and Noyes, 2011; Hung et al., 2018; Huang et al., 2020). To synthesize the studies concerning extended TAM models, Abdullah and Ward (2016) conducted a review study on the selection of the external variables and theorized the general extended TAM for e-learning (GETAMEL). However, there are few studies conducted to validate this model and verify whether the model can function as a rationale for follow-up research. Moreover, as pinpointed by the authors, the scope of their review is limited to the studies that “do not specify error values and only state significance levels” (Abdullah and Ward, 2016, p. 253), and therefore, the validity of the GETAMEL model and the conclusions reached based on this model may be problematic if the model *per se* is not sufficiently validated.

Due to the COVID-19 pandemic, college students in China had to proceed with their English as a foreign language (EFL) learning through various online learning systems developed by domestic textbook publishers. However, little is known about the technology acceptance of Chinese EFL learners faced with such an abrupt change toward the “new normal” of learning fully online (Herath and Herath, 2020). Therefore, the present study aims to validate the GETAMEL model by conducting a survey among EFL learners and examine the validity and reliability of the adapted instrument for the GETAMEL model.

In this study, there are two reasons for targeting foreign language learning as our research context: first, the nature of language learning is highly interactive (Canale and Swain, 1980) and peer interaction in foreign language classrooms is an indispensable way for learners to learn the target language (McCabe, 2017; Jiang et al., 2020). A complete shift of the learning mode due to COVID-19 may change the way language learners used to interact with one another in class and may further influence how students perceive the technology use in their online language learning. Second, the GETAMEL model is a general extended TAM model, and its applicability in a discipline-specific context needs to be validated. Testing the hypotheses formulated in a general model against the data obtained from a discipline-specific context is not only essential for a robust model but also may result in potential adjustments to revise the model for its better and broader use.

## Theory and Hypotheses

### Internal Constructs of the GETAMEL Model

The GETAMEL model was proposed as an extended TAM model and comprises two components: the internal constructs and five specified external factors. The internal constructs were known as the TAM model that was first proposed by Davis (1985) and has been verified and validated by enormous empirical studies ever since. In practice, the TAM model has evolved to become a core model in understanding the predictors of human behavior toward the potential acceptance or rejection of technology (Granić and Marangunić, 2019). Over the years, as it is considered as a robust, powerful, and parsimonious model, the TAM model has been widely used as a research framework to explain the technology acceptance of users in many studies under various contexts (Ursavaş, 2013). It is made up of two sorts of variables: (1) core variables of user motivation, including perceived ease of use (PEU), perceived usefulness (PU), and attitude toward technology (ATT) and (2) outcome variables such as behavioral intention (BI) to use the technology and actual use (AU).

**Figure 1:**
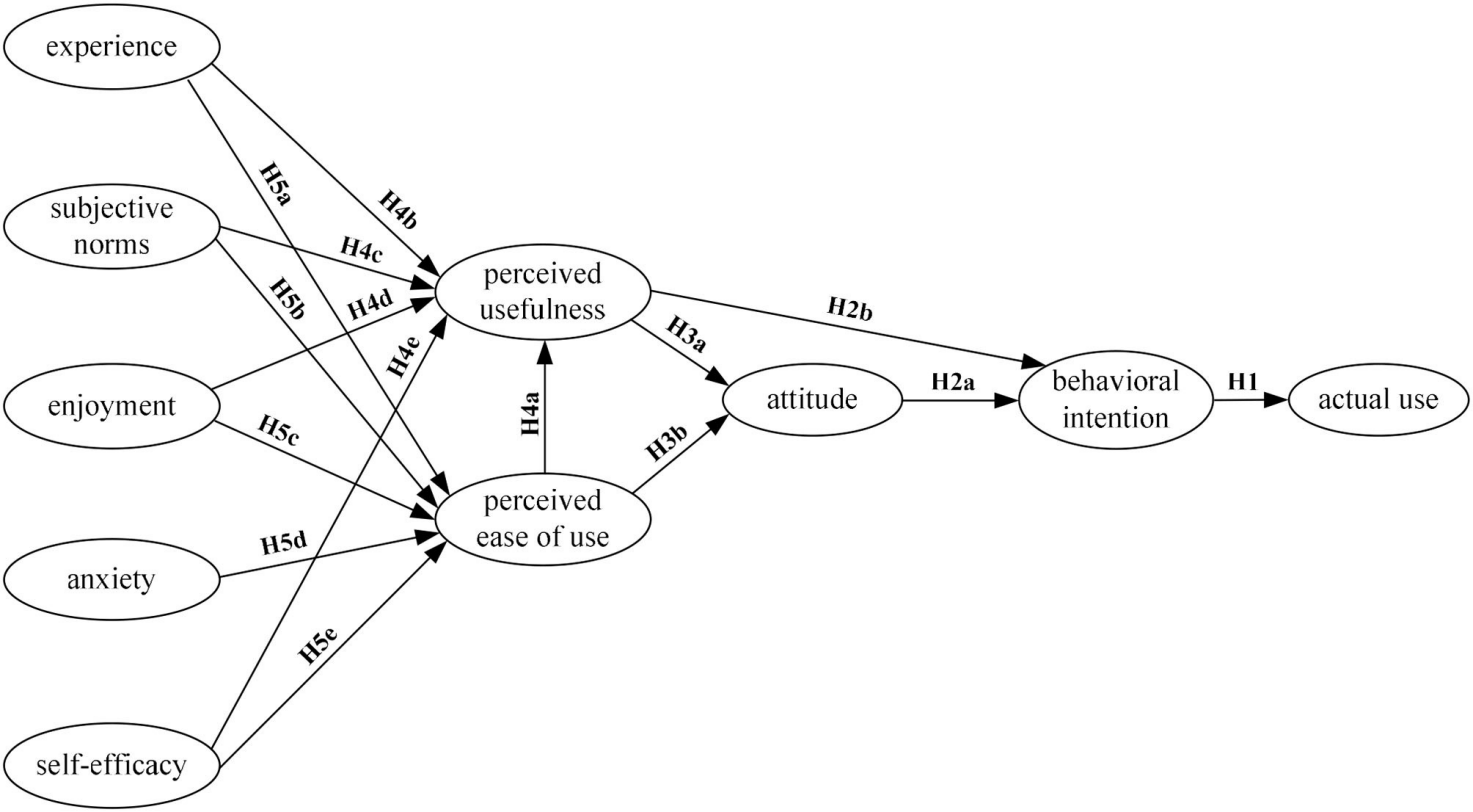
The general extended technology acceptance model for e-learning. *Source*: Abdullah and Ward (2016).

The internal TAM constructs were established following the idea from the theory of reasoned action (TRA) (Ajzen and Fishbein, 1980), which holds that the salient perceptions of people determine their attitude toward a stimulus object, and their attitude determines their intention to perform a certain behavior, and the intention will ultimately determine their actual behaviors (Agudo-Peregrina et al., 2014). Adapting the idea of TRA into the context of technology acceptance and use, Davis (1989) formulated the hypotheses that the acceptance or rejection of a specific technology by users, i.e., the AU of a specific technology, is fundamentally determined by PU and PEU (Marangunić and Granić, 2015), and this effect is mediated by the ATT of users and their BI to use the technology (as shown in Figure 1). The TAM constructs created a basis for understanding how external factors might influence the beliefs of people (i.e., PU and PEU), their attitude toward a given technology, their behavioral intention to use and their actual use of that technology (Park, 2009). Through the internal TAM constructs, researchers tend to understand and interpret how the perception of users on a new technology determines their intention to adopt or reject the technology and their actual technology use. By modeling this process, researchers and teachers can identify the potential adjustments that must be brought about by a new technology or system to make it more acceptable to users. For the past three decades, a range of issues were raised concerning the internal constructs, such as the debate over whether ATT is a necessary construct in the TAM model (e.g., Teo, 2009; Nistor and Heymann, 2010; López-Bonilla and López-Bonilla, 2011; Ursavaş, 2013).

As illustrated in Figure 1, AU is one outcome variable of observable behavior, whereas BI of users to use the technology is another outcome variable of intended behavior. It is, therefore, hypothesized that users' BI to use the technology is a direct determinant of their AU of the technology. Even though there is research pointing out that the direction of this relation is not deterministic as positive user experience may also predict future BI (Straub, 2009; Raza et al., 2021), yet in most cases, users' BI predicts their AU. Thus, in the context of online EFL learning, it is posited that:

*H1: Students' BI to use the online EFL learning system is positively related to their AU of the system*.

Attitude is defined as “the degree of evaluative affect that an individual associates with using the target system in his or her job” (Fishbein and Ajzen, 1975, p. 216). Adapting it to the present context of online EFL learning, we perceive ATT as the degree of evaluative affect with which students associate using the learning system in their fully online EFL learning. As hypothesized in the model, ATT positively predicts users' BI to use the technology, and in turn, it is partially influenced by users' PU, which is defined as “the degree to which a person believes that using a particular system would enhance his or her performance” (Davis, 1985, p. 26). Accordingly, we posit that:

*H2a: Students' ATT is positively related to their BI to use the online EFL learning system*.
*H2b: Students' PU of the online EFL learning system is positively related to their BI to use the system*.

Apart from PU, PEU, which is defined as “the degree to which a person believes that using a particular system would be free of effort” (Davis, 1985, p. 26), is another core construct in the internal TAM constructs. As claimed by Davis (1989), the two perception variables are hypothesized to jointly result in positive or negative ATT. Moreover, between the two core constructs, it is hypothesized that PEU is a determinant of PU, and the relation will not hold when reversed. This may be attributed to the assumption that “PU concerns the overall impact of technology use on job performance (process and outcome), whereas PEU pertains to the process of using the technology *per se”* (Teo and Noyes, 2011, p. 1646). Thus, adapting these hypotheses to the context of the present study, we posit that:

*H3a: Students' PU of the online EFL learning system is positively related to their ATT*.
*H3b: Students' PEU of the online EFL learning system is positively related to their ATT*.
*H4a: Students' PEU of the online EFL learning system is positively related to their PU of the system*.

### External Factors of the GETAMEL Model

The TAM model has gained great momentum in the field of educational technology in the past 30 years. However, it was found that the percentage variance explained in primary studies was merely around 40% (McFarland and Hamilton, 2006; Park, 2009), and the internal constructs did not fully reflect the specific influences of technological and usage-context factors that may alter the acceptance of users (Liu, 2010). In response, several researchers extended the TAM model and proposed that external factors influenced BI and AU through mediated effects on PU and PEU.

In extant studies with respect to the extended TAM models, some external variables (e.g., information and communication technology (ICT) self-efficacy, ICT anxiety, and prior experience of technology use) that directly influence PU and PEU are often investigated to further interpret the technology acceptance or rejection of users (e.g., Teo et al., 2008; Park, 2009; Liu, 2010; Teo and Noyes, 2011; Liu et al., 2019; Vladova et al., 2021). In these studies, the model was modified to check the external factors that had some influences on the acceptance of the technology in the learning environment. These external factors are also referred to as antecedents of the core internal constructs (i.e., PU and PEU). Through these external factors, researchers can identify some specific reasons for students accepting or rejecting to use a particular technology, according to which the course teachers may better integrate technology into course design and implementation. As argued by Legris et al. (2003), the internal TAM constructs without external factors can only provide broad information on the opinions of users about a particular technology.

To provide valuable insights into the relations between external factors and the internal constructs, some review studies and meta-analyses (e.g., Abdullah and Ward, 2016; Granić and Marangunić, 2019; Scherer et al., 2019; Al-Qaysi et al., 2020) were conducted. Among those synthesis studies, Abdullah and Ward (2016), in particular, investigated in detail the selection of external factors with regard to technology acceptance by students of e-learning systems. They analyzed 152 different external factors of the total of 107 empirical studies and identified some best predictors of the PEU and PU by the learners of e-learning systems. To be specific, they uncovered that four external factors, namely experience, subjective norm, enjoyment, and self-efficacy, were the best predictors of PU and PEU in the model. Additionally, for PEU, computer anxiety was identified as an extra predictor (as shown in Figure 1).

#### Experience

Several studies concluded that prior experience played a vital role in explaining the e-learning adoption and facilitating the adoption process (e.g., Gurung and Daniel, 2005; Al-alak and Alnawas, 2011; Hung et al., 2018; Alfadda and Mahdi, 2021). For example, experience can enhance the acceptance of new technology and temporarily help to reduce the anxiety and difficulties of adoption (Clough et al., 2008). In the review (Abdullah and Ward, 2016) experience was found to be one of the best predictors of both PEU and PU, and among the student subjects in the e-learning environment, the averaged effect size of both in terms of path coefficient (β) was 0.221 and 0.169, respectively. Although the review uncovered that the associations between experience and the two constructs were reported to be more non-significant (Abdullah and Ward, 2016), experience was still incorporated into the proposed model because of their salient effect size. Therefore, to test this relationship in online EFL learning environment, we posit that:

*H4b: Students' experience of online learning is positively related to their PU of the online EFL learning system*.
*H5a: Students' experience of online learning is positively related to their PEU of the online EFL learning system*.

#### Subjective Norm

Subjective norm refers to the degree to which an individual perceives that people who are important to him or her think he or she should (or should not) perform a behavior in question (Fishbein and Ajzen, 1975). Studies in educational and other fields have found that subjective norm has a significant and positive impact on the internal TAM constructs (e.g., Kumar et al., 2020). In the context of online learning amid the COVID-19 pandemic, students may be more ready to use some educational technologies during the lockdown when it is suggested or required by their peers, teachers, or any other influential people in their learning environment. Abdullah and Ward (2016) found that 86% of the reviewed studies reported a significant positive association between PU and subjective norm and 67% reported a significant positive association between PEU and subjective norm. The averaged effect size of subjective norm on the PU and PEU of students was found to be 0.301 and 0.195, which were considered small to medium and small, respectively (Cohen, 1988). Therefore, the paths between subjective norm and both PU and PEU were included in the GETAMEL. Thus, we posit that:

*H4c: Students' subjective norm is positively related to their PU of the online EFL learning system*.
*H5b: Students' subjective norm is positively related to their PEU of the online EFL learning system*.

#### Enjoyment

Enjoyment in learning is an important indicator of intrinsic motivation (Krapp and Prenzel, 2011; Huang et al., 2021), which is typically a positive, real-time emotion caused by ongoing learning activities. It reflects not only the extent to which learning is thought to be enjoyable and of intrinsic value, but also whether students regard themselves as capable. Following the definition of Park et al. (2012), in the context of the present study, enjoyment is considered as how enjoyable it is to use the particular online EFL learning system *per se*, excluding any performance consequences resulted from the system use. A few studies have found associations between enjoyment and the effective use of ICT by students (e.g., Pekrun et al., 2002). In their review, Abdullah and Ward (2016) reported a medium to large (Cohen, 1988) averaged effect size of enjoyment on students' PU (β = 0.452) and PEU (β = 0.341), and the associations between enjoyment, and PU as well as PEU were included in their model. Therefore, to test such relationships in our context, we posit that:

*H4d: Students' enjoyment in online learning is positively related to their PU of the online EFL learning system*.
*H5c: Students' enjoyment in online learning is positively related to their PEU of the online EFL learning system*.

#### ICT Anxiety

The term ICT anxiety is derived from previous studies on computer or technology anxiety. Computer anxiety refers to fears or concerns about the implications of computer use such as the loss of important data or other possible mistakes (Thatcher and Perrewé, 2002). Several empirical studies have concluded that computer or technology anxiety is associated with the avoidance or less use of computers and technology (e.g., Cazan et al., 2016; Kamal et al., 2020; Tsai et al., 2020). In the context of the present study, students used not only personal computers in online EFL learning but also used other mobile devices (e.g., tablets and mobile phones). Therefore, the broader term ICT anxiety was used instead of computer anxiety. According to the review (Abdullah and Ward, 2016), 59% of the studies reported a negative influence of computer anxiety on PEU. Among the student subjects, a negative association (β = −0.199) was found between their computer anxiety and PEU with a small to medium effect size (Cohen, 1988). However, the effect of computer anxiety on PU was found to be barely present (β = 0.070), and therefore, the association between computer anxiety and PU was excluded from the GETAMEL model. Therefore, we have only intended to test the association between ICT anxiety and PEU, and we posit that:

*H5d: Students' ICT anxiety is negatively related to their PEU of the online EFL learning system*.

#### ICT Self-Efficacy

Similar to ICT anxiety, the term ICT self-efficacy is also derived from previous studies on computer self-efficacy and due to the same reason mentioned above, we used the broader term ICT self-efficacy instead. ICT self-efficacy refers to the confidence of students in their computer- and internet-related ability to carry out specific tasks and it has been concluded in many reviews and empirical studies that ICT self-efficacy plays an integral role in a computer-mediated learning environment (e.g., Moos and Azevedo, 2009; Kumar et al., 2020; Pan, 2020; Zheng and Li, 2020). According to Abdullah and Ward (2016), among student subjects in an e-learning environment, self-efficacy had an averaged small to medium effect size on PU (β = 0.174) and an averaged medium effect size on PEU (β = 0.352) (Cohen, 1988). It was uncovered that 80% of the review studies found a statistically significant and positive effect of self-efficacy on PEU, and 63% indicated a lack of positive significant association between self-efficacy and PU. However, due to the magnitude of the effect size, self-efficacy was incorporated into the GETAMEL model as an external factor of both PU and PEU. Accordingly, to test such associations in our context, we posit that:

*H4e: Students' ICT self-efficacy is positively related to their PU of the online EFL learning system*.
*H5e: Students' ICT self-efficacy is positively related to their PEU of the online EFL learning system*.

Based on their review, Abdullah and Ward (2016) proposed the GETAMEL model with a completely bottom-up approach. Nonetheless, as these external factors were established in an inductive fashion, it may raise concerns when being applied in a domain- or discipline-specific context. Hence, the present study aims to test the validity of and the causal relationships among the latent variables established by the GETAMEL model. In particular, the present study is to examine whether those external factors summarized in the GETAMEL model can exert a statistically significant influence on the technology acceptance of students in a discipline-specific e-learning context (i.e., a fully online EFL course) amid the COVID-19 pandemic lockdown. To recap, the hypotheses proposed in this study were tabulated in Table 1 for an easier understanding by the readers.

**Table 1:** A summary of the hypotheses.

**GETAMEL components**	**Hypothesis**	**Path**
Internal constructs	H1	BI → AU
	H2a	ATT → BI
	H2b	PU → BI
	H3a	PU → ATT
	H3b	PEU → ATT
	H4a	PEU → PU
External factors	H4b	EXP → PU
	H4c	SN → PU
	H4d	ENJ → PU
	H4e	ICTSE → PU
	H5a	EXP → PEU
	H5b	SN → PEU
	H5c	ENJ → PEU
	H5d	ICTA → PEU
	H5e	ICTSE → PEU

*BI, behavioral intention; AU, actual use; ATT, attitude toward technology; PU, perceived usefulness; PEU, perceived ease of use; EXP, experience; SN, subjective norm; ENJ, enjoyment; ICTSE, ICT self-efficacy; ICTA, ICT anxiety*.

## Methods

### Context and Participants

In China, the earliest regional citywide lockdown policy took effect only one day before the Eve of Spring Festival (aka. the Chinese New Year), which is the biggest and most significant festival of the year for Chinese families. Therefore, millions of people could not return to their hometown to have their family reunion. In the whole 2020 Spring semester, most Chinese students at all levels (except for some students in their final year) were still observing the stay-at-home policy and learning completely online for the sake of health and safety considerations. Many teachers and students have been “forced” to conduct online teaching and learning for their first time in life. While online education has been growing in China in the past decade or so, it is the first time that the teachers and students in the whole nation completely replace traditional classroom with internet-based instructions.

A total of 678 undergraduate students majored in a range of subjects (i.e., Chinese literature and arts, education, mathematics, chemistry, biology, and computer science) from a university in China participated in the online questionnaire survey. Their average age was 18.3 years old; 42.1% of them were male and 57.9% were female. The study was approved by the university, and the students were well-informed of the purpose of the survey and gave their consent as participants before responding to the questionnaire formally. The participants were either in their first year or in their second year at the time of the survey and all registered “College English,” a compulsory EFL course for the students in Year 1 and Year 2. They reported an average score of 77.6 (out of 100) for their last term EFL course final exam, indicating that they had mostly met the course requirements and were eligible to continue with the course. According to their responses in the survey (after removing the invalid cases), 72.3% of the students had “never” or “seldom” participated in online English learning, and none of them had any experience of fully online English learning in their school education.

### Measures

The questionnaire consisted of eight major sections that assessed the external and internal variables. To ensure full understanding of the questionnaire by participants, all the 36 items were translated from English into Chinese. Backward translation was then used to make sure that each translated item was semantically equivalent to those of the original English version. With that, some items were revised in response to the current online EFL learning context. Two professors in the field of learning sciences were consulted for validating the items. Based on their advice adjustments in language expression were then made.

Perceived usefulness (four items), PEU (three items), and ATT (three items) were measured on a six-point Likert scale ranging from “*1* = *strongly disagree”* to “*6* = *strongly agree.”* These three subquestionnaires were adapted from the work of Tsai et al. (2020). After removing invalid data records, the Cronbach's α-values of the three subquestionnaires were 0.941, 0.760, and 0.919, respectively.

Subjective norm (six items), ICT anxiety (four items), and BI (three items) were also measured on a six-point Likert scale ranging from “*1* = *completely not true of me”* to “*6* = *completely true of me.”* The measure for subjective norm was adapted from the work of Huang et al. (2020), and the measures for ICT anxiety and BI were adapted from Tsai et al. (2020). After removing invalid data records, the Cronbach's α values of subjective norm, ICT anxiety, and BI were 0.835, 0.672, and 0.847, respectively. The reliability estimate of ICT anxiety is slightly less than the generally accepted threshold of 0.70, but according to Hair et al. (2010), Cronbach's α-values near 0.70 are still acceptable.

The measures for ICT self-efficacy (six items) and enjoyment (seven items) were both derived from Fraillon et al. (2014). ICT self-efficacy was scored on a six-point Likert scale ranging from “*1* = *I do not know how to do this”* to “*6* = *I know how to do this.”* Enjoyment was scored on a six-point Likert scale ranging from “*1* = *strongly disagree”* to “*6* = *strongly agree.”* After removing invalid data records, the Cronbach's α values for ICT self-efficacy and enjoyment were 0.857 and 0.868, respectively.

The experience was measured on one item, which is “Before the outbreak of this pandemic, what is your experience of having a fully online English course like the one we are having this semester?” This question was scored on a four-point Likert scale ranging from “*1* = *I have never had any experience of online English learning”* to “*4* = *I always participate in online English learning.”*

Actual use of the online EFL learning system was measured in terms of time spent by the students every day on the learning system for self-learning. They were required to estimate how much time they spent on the online learning system and choose among the seven options of time interval estimate. The options vary from “*1* = *Never”* to “*2* = *1–15 min every day”* to “*7* = *More than 90 min every day”* with an interval of 15 minutes each.

To minimize potential data contamination caused by careless respondents, we added three additional “filtering items” to the questionnaire. Three original items from the questionnaire were selected and paraphrased into three semantically identical statements. Then, they were paired up with the three original items. Thus, the six items constituted three semantic dyads, and each dyad possessed equivalence in meaning. Two professors in Chinese language and arts were consulted to ensure the semantic equivalence of each dyad. With that, the six items were placed back into the questionnaire. During the data screening process, if the response of a participant was deemed inconsistent (as shown in Section Data screening for specific filtering criteria) on the three dyads, we would remove it from the data set.

### Methods for Hypothesis Testing

The exploratory factor analysis (EFA) was first conducted with SPSS 25.0 to examine the construct validity of the external factors. Using Mplus 7, the confirmatory factor analysis (CFA) was then conducted to ensure the validity of the measurement model, and then the structural equation modeling (SEM) was performed to estimate all path coefficients (Asparouhov and Muthén, 2010). SPSS 25.0 and Excel were also used to produce descriptive statistics.

Typically, Chi-square (χ^2^), degree of freedom (*df*) together with the corresponding significance values (*p*), and other model fit information such as the comparative fit index (CFI), the Tucker-Lewis index (TLI), the root mean square error of approximation (RMSEA), and the standardized root mean square residual (SRMR) should be used to evaluate the model fit. Model fit is good when χ^2^/*df* is less than 3 and sometimes permissible when it is less than 5. Moreover, CFI and TLI should be no less than 0.95 for an excellent model fit and no less than 0.90 for an acceptable model fit (Huang et al., 2021). In addition, RMSEA and SRMR must be less than 0.06 and 0.08 for an excellent model fit, and 0.08 and 0.10 for an acceptable model fit (Schreiber et al., 2006).

## Data Analysis and Results

### Data Screening

During the preprocessing of the data, the responses of each participant on the three filtering dyads were calculated and compared, based on the results of which the decisions were made regarding whether a data record should be retained for analysis. The filtering criteria were: if the sum of the absolute value of the averaged difference between all dyads was >1 unit per dyad, then the responding performance of the participant was deemed inconsistent, and thus the corresponding data record was considered invalid for further analysis and should be excluded; otherwise, if the sum was ≤3, then the data record was retained for further analysis. By doing so, a total of 67 participants (9.88%) were removed from the sample, leaving 611 cases for further analysis. Because the questionnaire was administered online, the input checking mechanism of the system was set up automatically to verify each input, and thus there was no missing data or data in inappropriate format.

### Factor Analysis Results

The EFA was first performed to determine whether the items were properly loaded on four of the external factors (i.e., subjective norm, enjoyment, ICT anxiety, and ICT self-efficacy). The extraction method was principal axis factoring, and the rotation method was varimax. The EFA results showed that all the items were well-loaded on their corresponding constructs except for ICT anxiety. The factor loading of item 14 was 0.431, <0.5 (Hair et al., 2010) and item 23 was cross-loaded on at least two factors. Therefore, the two items were removed from ICT anxiety, and thus ICT anxiety was comprised of only two items (i.e., items 21 and 22). However, it is presumed that a factor consisting of only two items is prone to be unstable, and therefore, this is listed in the limitation section.

The CFA was then conducted to validate the constructs of the four external factors. The results showed that there seemed to exist some items with factor loadings <0.5 (Hair et al., 2010), causing the model fit not quite acceptable (χ^2^ = 723.675, *df* = 183, *p* < 0.001, CFI = 0.897, TLI = 0.881, RMSEA = 0.070, and SRMR = 0.052). Accordingly, model modification was conducted to covary error terms of three pairs of items that were part of the same factor. After model modification, the model fit indices of the measurement model became acceptable (χ^2^ = 586.255, *df* = 180, *p* < 0.001, CFI = 0.922, TLI = 0.909, RMSEA = 0.061, and SRMR = 0.051) (as shown in Table 2).

**Table 2:** CFA results of external factors and construct validity and reliability.

**External factors**	**Items**	**Factor loading**	**Cronbach's α**	**CR**	**AVE**
SN	15. My instructor thinks that the Internet is valuable for online English learning.	0.73	0.835	0.831	0.453
	16. My instructor's opinions are important to me.	0.67			
	17. My classmates think that using the Internet is valuable for online English learning.	0.77			
	18. My classmates' opinions are important to me.	0.64			
	19. My school is committed to supporting my efforts to use the Internet for learning.	0.60			
	20. The use of online learning is important in my university.	0.62			
ENJ	7. It is very important to me to work with a computer.	0.61	0.868	0.867	0.484
	8. I think using a computer is fun.	0.69			
	9. It is more fun to do my work using a computer than without a computer.	0.61			
	10. I use a computer because I am very interested in the technology.	0.66			
	11. I like learning how to do new things using a computer.	0.80			
	12. I often look for new ways to do things using a computer.	0.80			
	13. I enjoy using the Internet to find out information.	0.68			
ICTA	21. I feel apprehensive about using the online learning system.	0.57	0.670	0.703	0.553
	22. I hesitate to use the online learning system for fear of making mistakes that I cannot correct.	0.89			
ICTSE	How well can you do each of these tasks on a computer?				
	1. Search for and find a file on your computer;	0.63	0.857	0.861	0.510
	2. Edit digital photographs or other graphic images	0.62			
	3. Create or edit documents (e.g., assignments for school);	0.77			
	4. Search for and find information you need on the Internet;	0.75			
	5. Create a multimedia presentation (with sound, pictures, or video)	0.72			
	6. Upload text, images, or video to an online profile.	0.77			

*SN, subjective norm; ENJ, enjoyment; ICTA, ICT anxiety; ICTSE, ICT self-efficacy*.

For the internal constructs, CFA was also conducted to examine the measurement model. It was found that the model fit indices were acceptable (χ^2^ = 278.381, *df* = 59, *p* < 0.001, CFI = 0.969, TLI = 0.959, RMSEA = 0.078, and SRMR = 0.028), but the modification indices showed that two pairs of error terms need to be covaried. Then, the model fit became even better (χ^2^ = 209.115, *df* = 57, *p* < 0.001, CFI = 0.979, TLI = 0.971, RMSEA = 0.066, and SRMR = 0.022) (as shown in Table 3).

**Table 3:** CFA results of internal constructs and construct validity and reliability.

**Internal constructs**	**Items**	**Factor loading**	**Cronbach's α**	**CR**	**AVE**
PU	27. Using online learning system will improve my English learning.	0.87	0.941	0.936	0.785
	28. Using online learning system will make my English learning more convenient.	0.84			
	29. Using online learning system will make me more effective in English learning.	0.90			
	30. Overall, I find the online learning system to be useful in English learning.	0.94			
PEU	31. I find the online learning system to be clear and understandable.	0.90	0.760	0.743	0.503
	32. I find that the online learning system does not require a lot of mental effort.	0.50			
	33. I find the online learning system to be easy to use.	0.67			
ATT	34. I think that using the online learning system is a good idea.	0.88	0.919	0.918	0.788
	35. I think that using the online learning system is beneficial to me.	0.93			
	36. I have positive perception of using the online learning system.	0.85			
BI	24. If possible, I intend to use online learning system as a supplementary way to learn English.	0.78	0.847	0.846	0.648
	25. I will always try to use online learning system in my daily English learning.	0.77			
	26. If university continues to provide online English courses, I plan to use the online learning system frequently.	0.86			

*PU, perceived usefulness; PEU, perceived ease of use; ATT, attitude toward technology; BI, behavioral intention*.

Descriptive statistics showed that the means of all the variables showed no floor or ceiling effect (Table 4). Additionally, their magnitude of the skewness fell between 0.02 and 0.86, less than the generally accepted threshold of 1. Moreover, except for subjective norm whose kurtosis value (i.e., 2.24) was marginally higher than the threshold of 2.20 (Sposito et al., 1983), the kurtosis magnitude of the other variables fell between 0.15 and 2.09, all <2.20. The skewness and kurtosis indicated that the data are roughly normally distributed.

**Table 4:** Descriptive statistics.

**Variables**	**Mean**	**SD**	**Skewness**	**Kurtosis**
SN	4.38	0.74	−0.84	2.24
ENJ	4.41	0.67	0.15	2.09
ICTA	3.10	1.04	0.37	0.15
ICTSE	4.94	0.92	−0.59	−0.27
PU	4.20	0.99	−0.84	1.06
PEU	3.91	0.92	−0.49	0.80
ATT	4.22	0.99	−0.86	1.14
BI	4.07	0.99	−0.57	0.50
EXP	2.25	0.90	0.56	−0.38
AU	3.61	1.60	−0.02	−0.60

*n = 611; SN, subjective norm; ENJ, enjoyment; ICTA, ICT anxiety; ICTSE, ICT self-efficacy; PU, perceived usefulness; PEU, perceived ease of use; ATT, attitude toward technology; BI, behavioral intention; EXP, experience; AU, actual use*.

### Convergent and Divergent Validity

Furthermore, convergent validity and divergent validity were assessed to further validate the measurement models of external factors and the internal TAM constructs. Convergent validity and divergent validity are commonly regarded as the subsets of construct validity. Convergent validity tests that the possibly related constructs are, in fact, related, whereas divergent validity or discriminant validity tests that the constructs that are theorized to have no relationship do, in fact, not have any relationship.

According to Fornell and Larcher (1981), item reliability, composite reliability (CR), and the average variance extracted (AVE) were the three procedures to establish convergent validity. Item reliability was assessed by item factor loading onto the underlying construct. The factor loadings (Tables 2, 3) were all greater than the threshold of 0.50 (Hair et al., 2010), demonstrating acceptable convergent validity at the item level. On the other hand, at the construct level, CR and AVE are the two commonly employed indicators of convergent validity. As shown in Tables 2, 3, the CR-values of all the variables are acceptable (greater than the threshold of 0.70) (Hair et al., 2010). On the other hand, the AVE is a strict measure of convergent validity. As shown in Tables 2, 3, the AVE-values are all acceptable except for subjective norm and enjoyment, which are 0.453 and 0.484, marginally less than the threshold of 0.50 as suggested by Hair et al. (2010). Because the AVE is a more conservative indicator, according to Malhotra and Dash(2011, p. 702), “on the basis of CR alone, the researcher may conclude that the convergent validity of the construct is adequate.” Therefore, on an overall basis, the measurement model demonstrated adequate convergent validity.

Divergent validity is established when the measured constructs are, in fact, different. It can also be assessed at the item level and the construct level. Divergent validity is considered adequate when an item is correlated with the items that are loaded on the same construct more strongly than with those loaded on other constructs (Barclay et al., 1995). The inter-item correlation matrix revealed that the divergent validity on the item level for the four external factors was acceptable. For example, six items were loaded on ICT self-efficacy, and their inter-item correlation coefficients were no less than 0.416, whereas the inter-item correlation coefficients between the six items and those loaded on the other external factors were no greater than 0.276. Conversely, for the internal TAM constructs, the inter-item correlation coefficients of the items loaded on the same construct were mostly greater than those loaded on other constructs, but the within-construct inter-item correlation coefficients and between-construct inter-item correlation coefficients were all deemed quite high. Therefore, the divergent validity of the internal constructs might be marginally inadequate at the item level.

At the construct level, according to Hair et al. (2010), divergent validity is present when the variance that is shared between a given construct and the other constructs (i.e., inter-construct shared variance) in the model is less than the variance that the given construct shares with its measures (i.e., construct-measure shared variance). The inter-construct shared variance is the squared inter-construct correlation coefficient, and the construct-measure shared variance is equal to the AVE of the construct. When the inter-construct shared variance is less than the construct-measure shared variance, that is, the inter-construct correlation coefficient is less than the square root of the corresponding AVE value, and divergent validity is deemed to be adequate. Table 5 shows the correlation matrix between the variables and the square roots of their corresponding AVE values in parentheses. The square root of any AVE value in parentheses is much greater than the correlation coefficients in the same row or column, indicating good divergent validity of the external factors and the internal constructs.

**Table 5:** Inter-construct correlation matrix of the external factors and the internal constructs.

	**1**	**2**	**3**	**4**	**5**	**6**	**7**	**8**
1. SN	(0.673)							
2. ENJ	0.13^*^	(0.696)						
3. ICTA	0.11^*^	−0.03	(0.744)					
4. ICTSE	0.05^*^	0.05^*^	−0.06^*^	(0.714)				
5. PU	0.43^*^	0.15^*^	−0.03	0.07^*^	(0.886)			
6. PEU	0.39^*^	0.15^*^	−0.10	0.08^*^	0.82^*^	(0.783)		
7. ATT	0.41^*^	0.16^*^	−0.09	0.08^*^	0.83^*^	0.82^*^	(0.888)	
8. BI	0.41^*^	0.15^*^	−0.02	0.04	0.72^*^	0.70^*^	0.69^*^	(0.805)

*The square roots of AVE values are in parentheses on the diagonal; SN, subjective norm; ENJ, enjoyment; ICTA, ICT anxiety; ICTSE, ICT self-efficacy; PU, perceived usefulness; PEU, perceived ease of use; ATT, attitude toward technology; BI, behavioral intention*;

* *p < 0.001*.

### SEM Results

**Figure 2:**
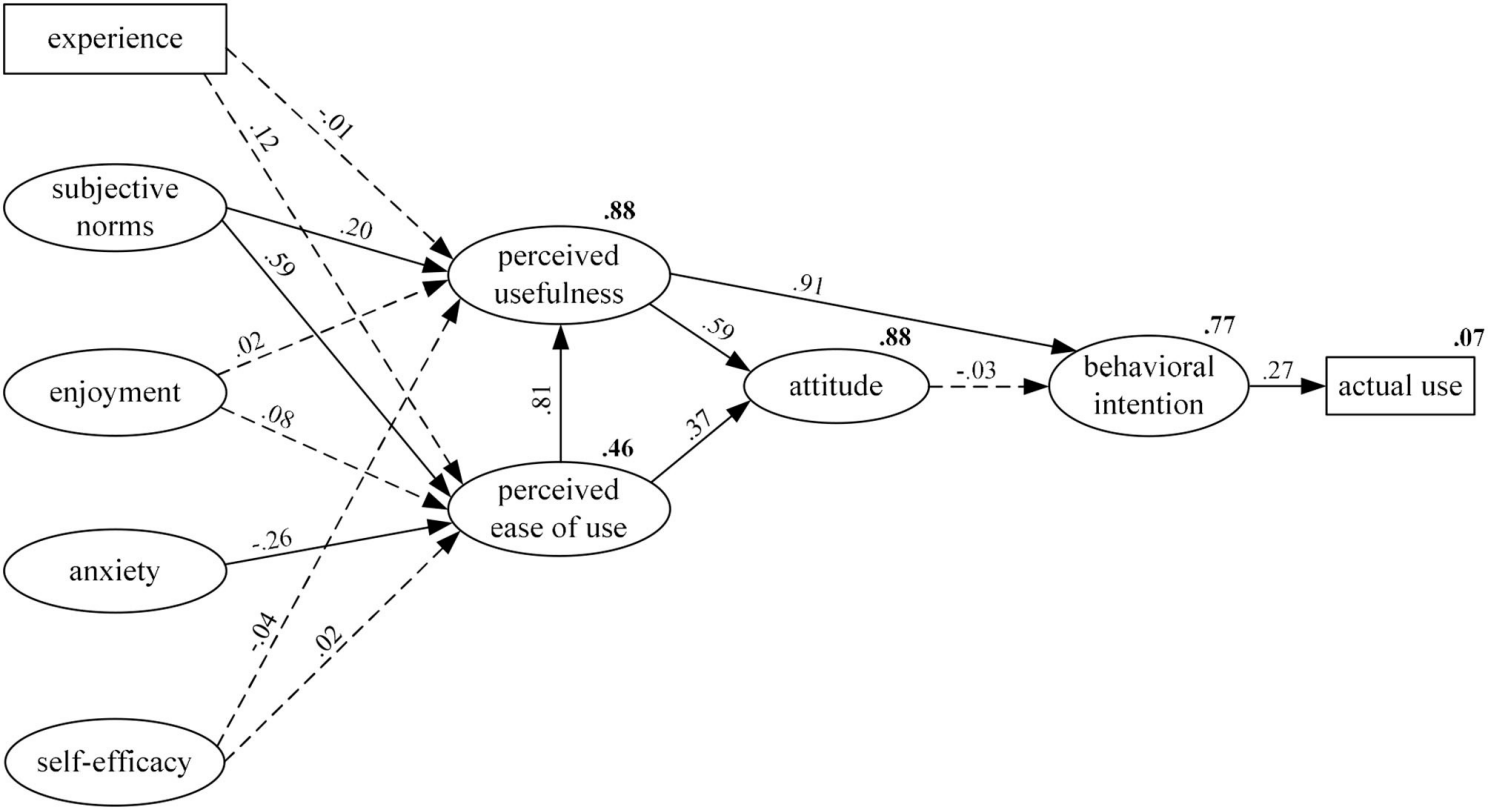
SEM results of the general extended technology acceptance model for e-learning. The numbers on each path were the standardized estimates of the path coefficients. All significant paths (significant at 0.001) are presented as solid lines and the non-significant paths are presented as dotted lines. The *R*^2^-values (variances explained) are labeled in bold to the top right of the internal constructs.

The SEM was conducted to test the model fit between the GETAMEL model and the data collected in the present study. Figure 2 reports the standardized path coefficients of the GETAMEL model and the squared multiple correlations (*R*^2^) of the endogenous variables. The model fit was found to be acceptable (χ^2^ = 1,664.658, *df* = 571, *p* < 0.001, CFI = 0.916, TLI = 0.908, RMSEA = 0.056, and SRMR = 0.053). The variance explained among the endogenous variables, i.e., the *R*^2^-values were of moderate to high magnitude (ranging from 0.46 to 0.88), except for AU, which was only 0.07, indicating that the relationship tested (BI → AU) might not be very meaningful because BI did not explain a sufficient variance in AU.

As shown in Figure 2, the hypotheses with regard to the internal constructs were all supported by our data except for ATT → BI. First, PEU had a significantly strong and positive influence on PU (β = 0.81, *p* < 0.001) and ATT (β = 0.37, *p* < 0.001), and PU was significantly related to ATT (β = 0.59, *p* < 0.001). Thus, H4a, H3b, and H3a were all supported. Second, PU had a significantly strong and positive influence on BI (β = 0.91, *p* < 0.001) while ATT was not significantly related to BI (β = −0.03, n.s.). Thus, H2b was supported but H2a was not. Third, BI is significantly and positively related to AU (β = 0.27, *p* < 0.001), and therefore, H1 was also supported.

For the external factors, only three out of the nine hypotheses were supported in our research context, and the other six were not. First, subjective norm was significantly and positively related to both PU (β = 0.20, *p* < 0.001) and PEU (β = 0.59, *p* < 0.001). Thus, H4c and H5b were both supported. ICT anxiety was found to be significantly but negatively related to PEU (β = −0.26, *p* < 0.001), and thus, H5d was also supported. Second, the association between experience and PU was non-significant and hardly present (β = −0.01, n.s.), and thus, H4b was not supported. Experience was found to be positively related to PEU (β = 0.12, *p* = 0.002) on the significant level of 0.01, but on a 0.001 level, it is not significantly related to PEU. Thus, H5a was not supported. Third, enjoyment was not found to have a significant influence on either PU (β = 0.02, n.s.) or PEU (β = 0.08, n.s.). Likewise, ICT self-efficacy was not significantly related to either PU (β = −0.04, n.s.) or PEU (β = 0.02, n.s.). Thus, none of the H4d, H5c, H4e, and H5e were supported in our context. A summary of the hypothesis testing results is shown in Table 6.

**Table 6:** Hypothesis testing results.

**GETAMEL components**	**Hypothesis**	**Path**	**β-value**	**Result**
Internal constructs	H1	BI → AU	0.27^**^	Supported
	H2a	ATT → BI	−0.03	Not supported
	H2b	PU → BI	0.91^**^	Supported
	H3a	PU → ATT	0.59^**^	Supported
	H3b	PEU → ATT	0.37^**^	Supported
	H4a	PEU → PU	0.81^**^	Supported
External factors	H4b	EXP → PU	−0.01	Not supported
	H4c	SN → PU	0.20^**^	Supported
	H4d	ENJ → PU	0.02	Not supported
	H4e	ICTSE → PU	−0.04	Not supported
	H5a	EXP → PEU	0.12	Not supported
	H5b	SN → PEU	0.59^**^	Supported
	H5c	ENJ → PEU	0.08	Not supported
	H5d	ICTA → PEU	−0.26^**^	Supported
	H5e	ICTSE → PEU	0.02	Not supported

** *p < 0.001; n = 611; SN, subjective norm; ENJ, enjoyment; ICTA, ICT anxiety; ICTSE, ICT self-efficacy; PU, perceived usefulness; PEU, perceived ease of use; ATT, attitude toward technology; BI, behavioral intention; EXP, experience; AU, actual use*.

## Discussion

Using the data collected from a fully online EFL course, the present study validated the GETAMEL model proposed by Abdullah and Ward (2016) and tested the related hypotheses. It was found that the hypotheses with regard to the internal constructs were mostly supported, whereas only one-third of the hypotheses concerning the external factors were supported. The results raised some serious concerns of construct validity over the external factors established in the GETAMEL model. To be specific, both enjoyment and ICT self-efficacy were not significantly related to either PU or PEU, and experience was found to be only weakly related to PEU on a 0.01 level and not significantly related to PU. As for the internal constructs, the ATT of students did not mediate between their PU of the online EFL learning system and their BI to use it (because of a non-significant association between ATT and BI) even though most studies uncovered that ATT was a mediator of the influence of PU on BI.

### Limited Role of Attitude Towards Technology

Inconsistent with most studies demonstrating a significant effect of ATT on the impact of users' PU on their BI to use the system (e.g., Teo et al., 2008; Huang et al., 2020), the present study found that there was no such mediating effect. The results showed a significantly direct effect of PU on BI, which was positive and strong (β = 0.91, *p* < 0.001). This direct effect was found to be so strong that it may have caused the indirect effect on BI to be non-significant (*p* = 0.771), indicating that ATT may play a limited role in the TAM model. This finding is echoed by some studies regarding ATT in TAM studies (e.g., Davis et al., 1989; Venkatesh, 2000; Teo, 2009). By comparing the original TAM model with a revised parsimonious TAM without ATT (Davis et al., 1989), based on empirical data, it was found that the role of ATT in explaining BI or AU was quite limited, and was “at best a partial mediator for the relationship between salient beliefs and user acceptance” (Kim et al., 2009, p. 67). Teo and Noyes (2011) also reported that ATT contributed only modestly to the TAM. Ursavaş (2013) also found that although it played a significant role as a predictor of BI to use technology, ATT did not contribute to the overall variance in usage.

Davis et al. (1989) explained the limited role of ATT as originating from people intending to use a technology because it was useful even though they did not have a positive attitude toward a particular technology. This explanation is very much in line with our research context. Due to the COVID-19 pandemic, the students all were studying at home through e-learning systems. Even though they might not hold a positive attitude toward the technology involved in their online learning, they had to use it anyway because it was the only way at the moment. Nistor et al. (2019) also pointed out that the attitude strength of students might be more of a construct to be integrated into TAM models rather than ATT. To some extent, the students might be “forced” to admit that the e-learning system during the lockdown is useful to their studies regardless of what their true attitude was toward the system. As argued by Davis et al. (1989), the absence of ATT in the model could help to better understand the effects of PU and PEU on the outcome variable of intended behavior to use the technology. Such an argument was also echoed by our data because with no mediating effect of ATT on the influence of PU on BI, the direct effect of PU on BI was found to be remarkably strong in the GETAMEL model.

### A General Model in a Specific Context

Abdullah and Ward (2016) identified and integrated the five most commonly investigated external factors into their GETAMEL model. Nevertheless, the present study revealed that two-thirds of the hypotheses concerning the external factors were not supported by the data. Specifically, enjoyment was found to have no significant influence on either PU or PEU. This finding is quite contradictory to what has been learned about the positive role of enjoyment in TAM studies (e.g., Teo and Noyes, 2011). However, some researchers did report a non-significant effect of enjoyment on internal TAM constructs in their studies. For example, Park et al. (2012) investigated the acceptance of web-based training by construction professionals and found that enjoyment had no significant effect on PEU. Brown et al. (2006) also found that perceived enjoyment did not significantly predict PEU. Those studies, however, merely reported the findings with no attempts made to explore the potential reasons. The present study was conducted in an EFL context, and as evident, interaction is particularly critical in a foreign language class (Jiang et al., 2020). However, due to an abrupt shift from regular classroom to online learning, internet-based peer interaction may be more difficult than that in a face-to-face mode. Besides, network transmission delay and the instability of hardware may greatly increase students' negative experience during their whole-semester online learning. Therefore, the students might not feel the joy of online EFL learning. Instead, they might have reduced the acceptance of technology because of the semester-long use of unsatisfactory online learning. As concluded by Ku and Lohr (2003), students might be ambivalent about taking online courses, but due to the COVID-19 lockdown, they had no other choice. Probably due to this special condition, enjoyment as an external factor did not significantly predict PEU and PU.

The local Chinese culture may also explain the limited role of enjoyment in the GETAMEL model. Peer interaction was considered a major source of enjoyment by EFL learners (Jiang and Dewaele, 2019). However, as Chinese EFL education has long been considered an examination-oriented system (focusing on reading and writing heavily), students seldom have the chances to communicate with each other in English orally (Amoah and Yeboah, 2021). Furthermore, Chinese culture advocates the readiness of learners to conform to school authorities (Lee and Yin, 2011), and Chinese students are likely to be obedient and passive learners who participate less actively in the classroom (Yan and He, 2020). Therefore, they are not accustomed to interacting with their peers in EFL classrooms. Additionally, under the influence of the COVID-19 pandemic, learning in front of a computer screen individually resulted in a less learner-friendly learning atmosphere than regular face-to-face classrooms for language learners to interact freely. Therefore, enjoyment was not significantly associated with the internal constructs as an external factor. Similarly, ICT self-efficacy was also found to be a non-significant external factor in GETAMEL in the present study, even though extant studies have shown that ICT self-efficacy plays a critical role in influencing users' PU and PEU (e.g., Park, 2009; Liu, 2010; Teo, 2018). As pinpointed in the review (Abdullah and Ward, 2016), 63% of the studies indicated a lack of significant association between self-efficacy and PEU. However, the authors still determined to include self-efficacy as an antecedent. Their inclusion criterion was utterly and simplistically determined by the frequency of the external factors investigated in the studies reviewed, which was bound to raise some theoretical concerns in the model *per se*.

The two external factors (i.e., enjoyment and ICT self-efficacy) did not have significant effects on the internal constructs, which may be attributed to the “general” orientation of the model. Evidently, the GETAMEL model was a broad model that did not take into account the characteristics of specific pedagogical contexts. Different classroom learning contexts may require different external factors to explain learners' technology acceptance and use. For example, students may still do well in lecture-based virtual mathematics classrooms, but foreign language classes must provide students with as many opportunities for interaction as possible (Peterson, 2009; Jiang et al., 2020). Additionally, due to the COVID-19 situation, the students were provided with no other choices but had to use the system regardless of their ICT self-efficacy and their perceived enjoyment in online learning. Therefore, the two external factors cannot significantly influence the BI of students and their AU of the system. While it is imprudent to directly eliminate the two external factors in the GETAMEL model, yet more empirical studies and discussions are needed before we can determine whether this general model should be further revised.

### Experience as an Antecedent or a Moderator?

In the GETAMEL model, the experience was the fifth commonly investigated variable selected as an external factor and its averaged effect size was small to medium (Abdullah and Ward, 2016). However, when we examined the tabulated data provided by the authors, we found that among student subjects, 67.7% and 75.0% of the studies reported a non-significant effect of experience on PU and PEU, respectively (Abdullah and Ward, 2016). The present study obtained the similar results: experience was found to have no significant effect on either PEU or PU (its effect on PEU was only significant at a 0.01 level), indicating a very limited effect. The contradictory and inconsistent effect may also result from the simplistic framing of the GETAMEL model. According to the study of Hung et al. (2018) on the acceptance of e-textbooks, experience had a significant moderating effect in their extended TAM model, and for experienced and inexperienced users, the conclusions greatly differed. Therefore, as some extant studies have revealed (e.g., Castañeda et al., 2007; Hsieh and Liao, 2011), it may make more sense to revise the GETAMEL model and integrate experience as a moderator rather than as an antecedent.

In the present study, most of the students surveyed had little experience of formal online learning, and therefore, more evidence is needed to confirm whether experience should be integrated as an antecedent or a moderator. However, from the perspective of a system developer, this may be a desirable result, “as it suggests that the use of a well-designed e-learning system does not depend on previous internet experience or self-efficacy” (Pituch and Lee, 2006, p. 238). Moreover, to further test the effect of experience in the GETAMEL model, the measurement of experience may be a matter of significance because many studies still reply on self-reported data to evaluate the prior experience and even future system use of a user (Agudo-Peregrina et al., 2014).

## Conclusion, Limitations, and Implications

Using the data collected from an online EFL course during the COVID-19 lockdown, the present study validated the GETAMEL model proposed by Abdullah and Ward (2016). The findings were three-fold: (1) While it could be significantly predicted by PEU and PU, ATT did not mediate between PU and BI, indicating that ATT played a very limited role among the internal constructs in the GETAMEL model; (2) Enjoyment and ICT self-efficacy had no significant effect on PU and PEU, which raised concerns on the applicability of the general model into a specific context; and (3) Experience was included in the GETAMEL model as an antecedent but our data had contradictory results. To be specific, at a 0.001 level, experience was not a significant antecedent of the internal constructs in the GETAMEL model.

One major limitation of the present study is the representativeness of the sample. The participants were only enrolled in one university in China. Therefore, more studies in different tertiary online EFL settings are needed to explore students' technology acceptance during the COVID-19 outbreak. Another limitation is the assessment of students' AU of online learning system. Because the survey was administered anonymously, the data gathered could not be matched with their behavioral data from the online learning system. Therefore, this study could only use self-reported data to evaluate participants' AU of the system, which might have resulted in systematic errors in assessment. As far as the analysis result was concerned, the *R*^2^-value of AU was only 0.07, indicating that over 90% of the variance in AU was not properly explained by the data. Third, the measure of ICT anxiety was only comprised of two items, and future studies may need to consider revising the item expressions or adapting a different instrument to measure it adequately.

A theoretical implication of the present study is that the external factors integrated into the GETAMEL model were found to fit the specific learning context poorly, indicating that a model modification is needed for the external factors in the GETAMEL model. External factors such as experience may be integrated as a moderator rather than as an antecedent. As mentioned before, it would be controversial and problematic when the inclusion criterion of the external factors was simply based on how many studies had investigated a particular variable. To enhance the robustness of the GETAMEL model, future studies need to include domain- or discipline-specific variables into this model to surface the impact of disciplinary characteristics on users' technology acceptance. On the other hand, the present study found that the role of ATT might not be a mediating variable under the influence of the COVID-19 lockdown. Accordingly, in practice, students' ATT may be revisited under such a circumstance when EFL teachers design online course activities. A proper understanding of students' attitude toward the technologies employed in a fully online classroom under the influence of COVID-19 may improve the learning performance of students. Moreover, future studies may need to consider more demographic information of the participants such as socioeconomic status and relevant cultural factors in understanding the relationships between the external factors and the internal constructs.

## Data Availability Statement

The raw data supporting the conclusions of this article will be made available by the authors, without undue reservation.

## Ethics Statement

The studies involving human participants were reviewed and approved by Local Ethics Committee of The Chinese University of Hong Kong. The patients/participants provided their written informed consent to participate in this study.

## Author Contributions

MJ made contributions to the conception or design of the work, analysis or interpretation of data for the work, and drafting the work. MC made comments on the content and also proofread the manuscript. C-sC agreed to be accountable for all aspects of the work in ensuring that questions related to the accuracy or integrity of any part of the work are appropriately investigated and resolved. Y-lM collected the data. MJ and WL revised the manuscript critically. MJ also provided approval for publication of the content. All authors contributed to the article and approved the submitted version.

## Conflict of Interest

The authors declare that the research was conducted in the absence of any commercial or financial relationships that could be construed as a potential conflict of interest.

## Publisher's Note

All claims expressed in this article are solely those of the authors and do not necessarily represent those of their affiliated organizations, or those of the publisher, the editors and the reviewers. Any product that may be evaluated in this article, or claim that may be made by its manufacturer, is not guaranteed or endorsed by the publisher.
